# Intrapancreatic Accessory Spleen Diagnosed As Neuroendocrine Tumor: The Dangers of False Positives and Their Implications in Subsequent Management

**DOI:** 10.7759/cureus.15891

**Published:** 2021-06-24

**Authors:** Simran Kripalani, Vikram Patel, Upasana Joneja, Shikha Talwar, Meet Parikh, Veniamin Barshay, Adib Chaaya

**Affiliations:** 1 Department of Gastroenterology, Cooper Medical School of Rowan University, Camden, USA; 2 Department of Gastroenterology, Cooper University Hospital, Camden, USA; 3 Department of Pathology, Cooper University Hospital, Camden, USA; 4 Department of Medicine, Cooper University Hospital, Camden, USA; 5 Department of Radiology, Cooper University Hospital, Camden, USA

**Keywords:** splenosis, accessory spleen, pancreatic neuroendocrine tumor, net, pet-ct netspot

## Abstract

This case serves as a reminder to consider ectopic splenic tissue in the differential diagnosis of pancreatic masses. The literature shows a lack of awareness and overtreatment of this condition due to clinical and radiologic concern for malignancy, namely neuroendocrine tumors (NETs) identified on positron emission tomography (PET)-CT NETSPOT. Given the vast difference in management and prognosis of ectopic splenic anomalies and malignant neoplasms involving the pancreas, accurate diagnosis is imperative to avoid unnecessary invasive procedures such as Whipple or distal pancreatectomy and splenectomy, which are associated with increased morbidity and mortality.

## Introduction

Splenosis and accessory spleen are both ectopic splenic anomalies. Splenosis or autotransplantation of splenic tissue occurs after trauma or surgery in locations outside of the spleen, most commonly in the abdominal and pelvic cavity [1]. The accessory spleen is a congenital anomaly of the spleen which results from the imperfect fusion of separate splenic masses during embryonic development. It has an incidence of 10-30% in the population and the most common sites include splenic hilum (~80%) and pancreatic tail (~20%) [2,3]. While the etiologies of both anomalies are distinct, their presentations are often mistaken for neoplasms of the primary organ they are found in. Intrapancreatic location of ectopic splenic tissue may mimic primary and secondary pancreatic malignant neoplasms such as pancreatic neuroendocrine tumor (NET), acinar cell carcinoma, ductal adenocarcinoma, and metastatic carcinoma [4-6]. In order to avoid unnecessary pancreatic resections and invasive procedures, it is important to correctly diagnose and manage pathologies involving the pancreas [7].

## Case presentation

**Figure 1:**
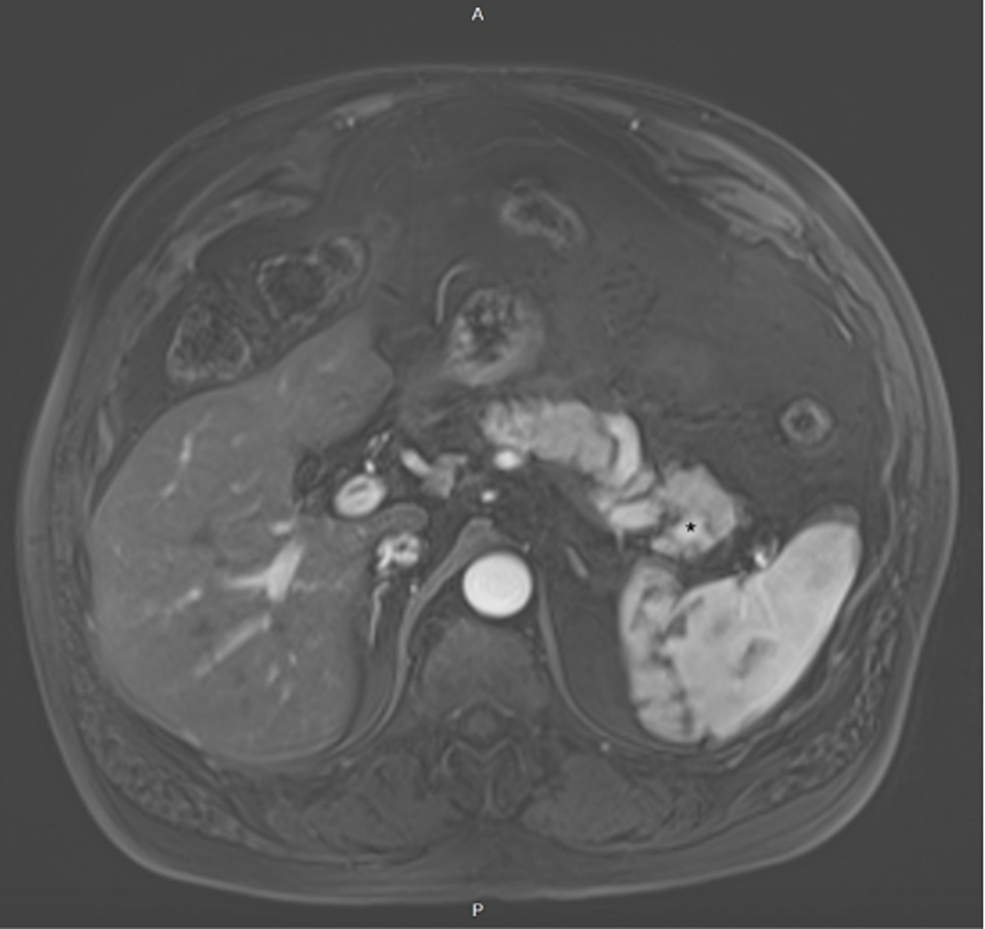
MRI, axial T1 fat-saturated post-contrast image with a pancreatic tumor. Pancreatic tumor marked (*)

**Figure 2:**
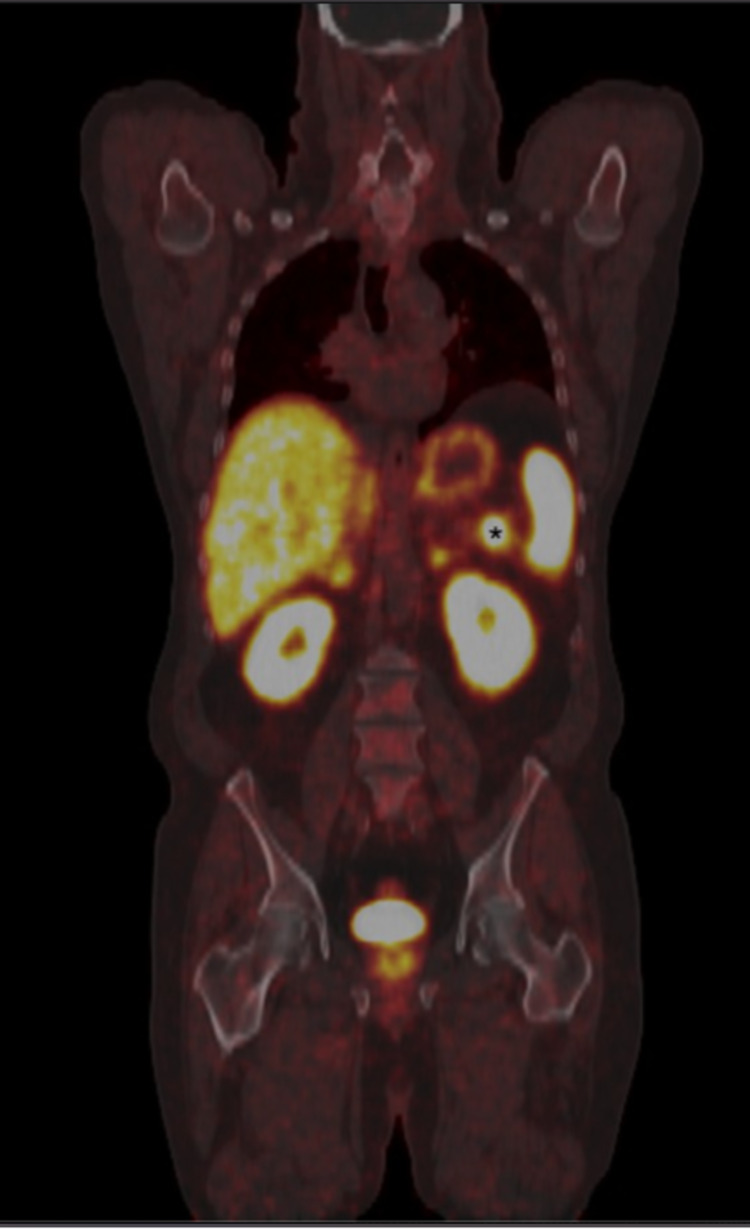
PET-CT NETSPOT, coronal view, with intrapancreatic tumor. Intrapancreatic tumor marked with (*) PET: Positron emission tomography

**Figure 3:**
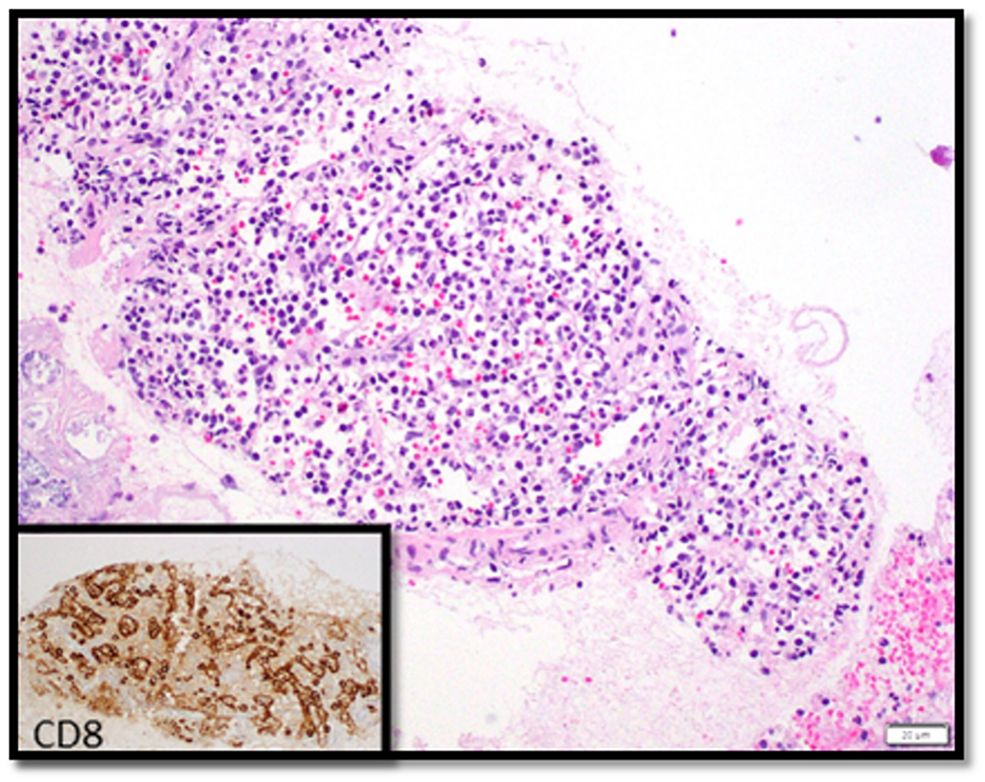
Pathology of pancreatic tail mass fine needle aspiration showing benign splenic tissue. CD8 highlights splenic sinusoidal cells in the secondary image.

A 70-year-old male with a past medical history of melanoma, skin squamous cell carcinoma of the scalp, and gastroesophageal reflux disease presented for chronic vague epigastric discomfort and bloating associated with intermittent bilateral flank pain which was present intermittently since his cholecystectomy 12 years ago. He also endorsed weight loss due to poor oral intake associated with an increase in abdominal girth. Physical exam showed a non-distended abdomen with diffuse tenderness and normal bowel sounds. He underwent an upper GI endoscopy that revealed mild chronic inactive gastritis without Helicobacter pylori (H. pylori) organisms. A concurrent colonoscopy showed multiple adenomatous polyps and diverticulosis. Despite proton pump inhibitor therapy, his symptoms persisted, and an MRI abdomen/pelvis was obtained (Figure 1). Imaging revealed a 1.7 cm x 1.2 cm pancreatic tail mass which was isointense to the spleen and pancreas with prominent solid arterial phase enhancement and moderate diffusion restriction suggestive of an NET without any evidence of abdominal metastatic spread. Chromogranin A level was 249.6 (normal 0-101.8 ng/mL). Subsequently, an upper endoscopy ultrasound (EUS) with fine needle aspiration (FNA) was performed, revealing a poorly defined irregular mass. The cytology specimen was non-diagnostic; it comprised of benign pancreatic parenchyma, lymphocytes, eosinophils, fibrous tissue, and degenerated cells. In an attempt for further characterization of the mass, a positron emission tomography (PET)-CT NETSPOT was performed (Figure 2). This showed radiotracer accumulation in the pancreatic tail mass, compatible with somatostatin receptor (SSTR) avid tumor suggestive of a NET without evidence of metastatic disease. With a high pre-test probability of NET, a repeat EUS with FNA was performed and cytology findings showed benign splenic tissue and pancreatic acinar cells without evidence of malignancy (Figure 3). Given the overall findings, the accessory spleen was the final diagnosis for the pancreatic tail mass. No further intervention was required. 

## Discussion

PET-CT NETSPOT received FDA approval in 2016 for functional imaging detection of SSTR avid NETs due to its high sensitivity in detecting small-sized tumors compared to the previously utilized octreotide scan [5,8,9]. PET-CT NETSPOT involves the injection of Ga-68 dotatate (NETSPOT) to target SSTRs that are present on the majority of NETs, at least well-differentiated types [9,10]. With a sensitivity of 96%, specificity of 93%, the accuracy of 94%, the positive predictive value of 96%, the negative predictive value of 93%, and additional benefits of less pre-procedure preparation, and less radiation exposure, it has become a new reference test in imaging assessment, and management of NETs [10]. Despite such high specificity and sensitivity, false positives are known to occur mostly due to the presence of SSTRs in other organs such as the spleen, native pancreatic uncinate process, pituitary gland, adrenal gland, kidney, and urinary bladder. False-positive PET-CT NETSPOT has also been seen with some pathologic processes such as osteoblastic osseous processes (degenerative joint disease, fibrous dysplasia, osseous hemangioma), inflammatory processes in lymph nodes, and other tumors such as meningiomas and pheochromocytomas [11].

Literature documents many case reports of false positives on PET-CT NETSPOT due to ectopic splenic anomalies [12-17]. One case report with a literature review showed that 66.7% of patients with intrapancreatic accessory spleen ended up undergoing unnecessary pancreatic resections [13]. With advanced imaging techniques and early detection, fear of overtreatment of this benign incidental process exists [18,19].

This case highlights the importance of considering ectopic splenic anomaly in the differential diagnosis of pancreatic masses that are small, 1-3 cm in size, located in the pancreatic tail, and are positive on PET-CT NETSPOT. Certain features on CT and MRI can suggest the diagnosis of intrapancreatic accessory spleen over pancreatic tumors. CT and MRI show attenuation and enhancement of the accessory spleen similar to the spleen in all phases. In contrast, pancreatic tumors show greater attenuation in the arterial phase and less in the venous phase on CT. On MRI, splenic tissue in the pancreatic tail often shows low signal intensity in T1 and high intensity in T2 when compared to pancreatic parenchyma; moreover, intratumoral hemorrhage and necrosis are absent in splenic tissue and may be seen with neoplasms [20]. In difficult cases, Technetium Tc 99m sulfur colloid scintigraphy can be utilized in differentiating splenic tissue from pancreatic tumors. This test uses Technetium-99m heat-damaged RBCs that have positive uptake in splenic tissue but not in NETs. While this technique could have been utilized for our patient, a biopsy was performed in lieu of other available techniques. A study by Liberini et al. showed that utilization of motion correction factors in PET/CT and high standard uptake value (SUV) cut-off (suggested at 42) can also help distinguish NETs from ectopic splenic anomalies [15].

## Conclusions

Ectopic splenic anomalies should be considered in the differential diagnosis of pancreatic masses that are small, 1-3 cm in size, located in the pancreatic tail, and are positive on PET-CT NETSPOT. Ectopic splenic anomalies are incidental, asymptomatic benign processes that do not need surgical intervention. Unfortunately, due to misdiagnosis and concern for malignant tumors such as NETs, patients undergo unnecessary procedures and surgeries. If this differential diagnosis is considered, additional imaging techniques such as Technetium-99m heat-damaged RBC or sulfur colloid scans can be helpful. Alternatively, tissue diagnosis may be performed, such as in this case, before committing to surgical resection. While FNA procedure-related complications and false negatives can occur, in our patient’s case, the biopsy provided the correct diagnosis and saved the patient unnecessary surgery, treatment, and anxiety.
